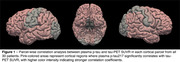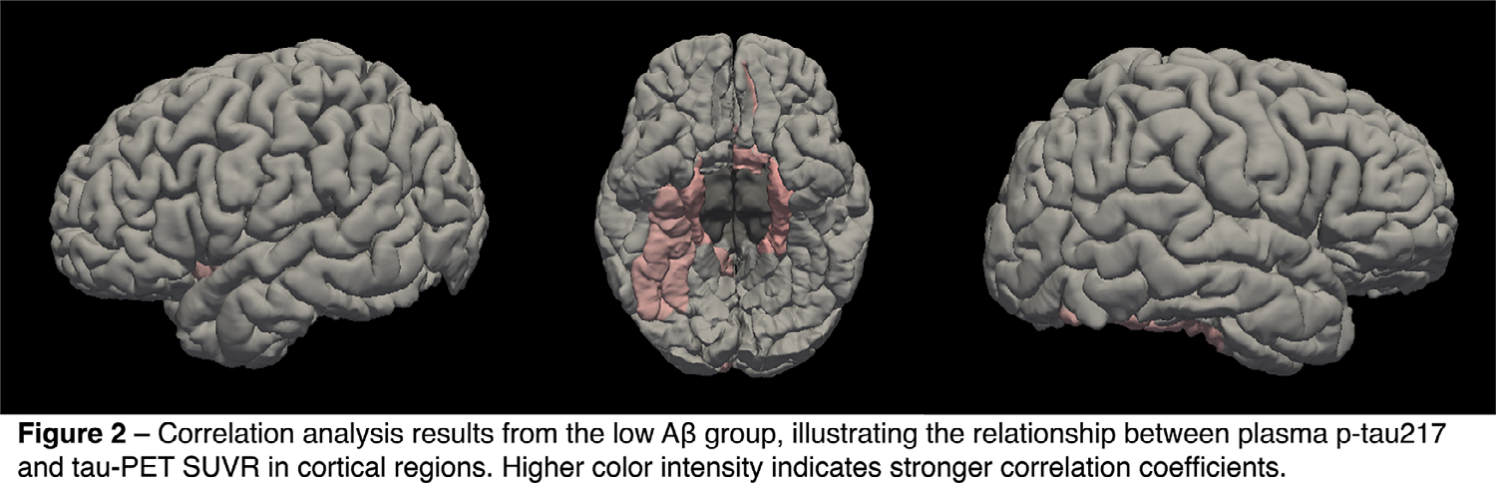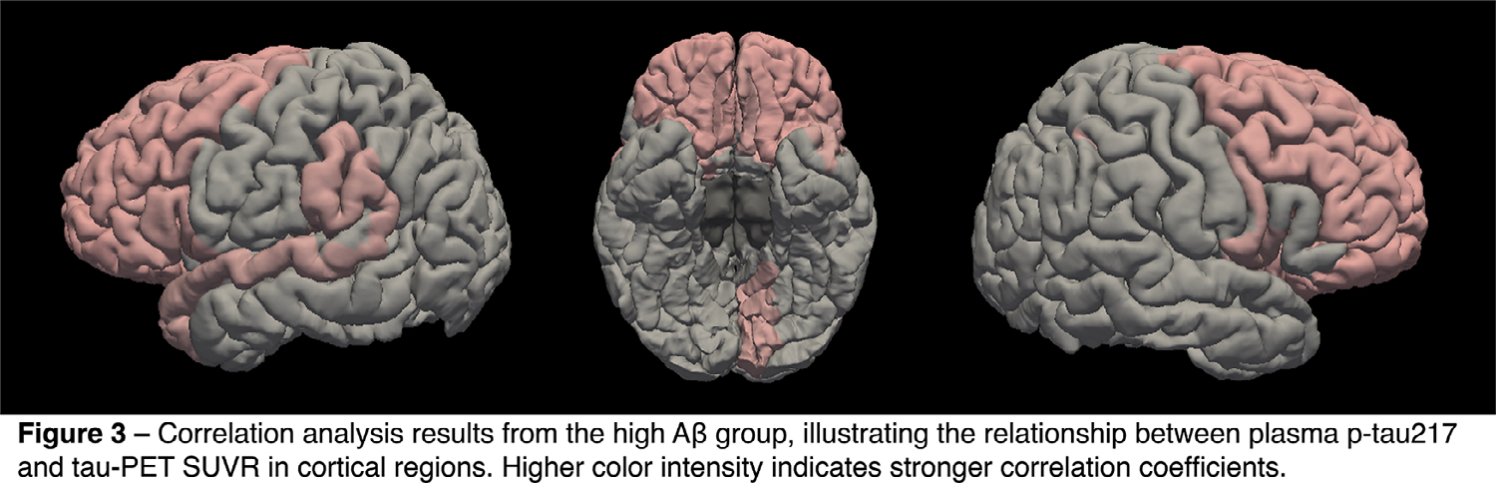# Relationship Between Plasma Phosphorylated Tau and Tau‐PET Spatial Patterns in Thai Patients with Amnestic Mild Cognitive Impairment or Mild Dementia Due to Probable Alzheimer's Disease: A Parcel‐wise Analysis

**DOI:** 10.1002/alz.088888

**Published:** 2025-01-09

**Authors:** Sekh Thanprasertsuk, Setthanan Jarukasemkit, Kittithatch Booncharoen, Watayuth Luechaipanit, Thanaporn Haethaisong, Adipa Chongsuksantikul, Thirawat Supharatpariyakorn, Yuttachai Likitjaroen, Thiravat Hemachudha, Chaipat Chunharas, Poosanu Thanapornsangsuth

**Affiliations:** ^1^ Faculty of Medicine, Chulalongkorn University, Bangkok Thailand; ^2^ Cognitive, Clinical and Computational Neuroscience (CCCN) Center of Excellence, Chulalongkorn University, Bangkok Thailand; ^3^ Chula Neuroscience Center, King Chulalongkorn Memorial Hospital, Bangkok Thailand; ^4^ Memory Clinic, King Chulalongkorn Memorial Hospital, Bangkok Thailand; ^5^ Faculty of Medicine Ramathibodi Hospital, Bangkok Thailand; ^6^ Neurocognitive Unit, Division of Neurology, Faculty of Medicine, Chulalongkorn University, Bangkok Thailand; ^7^ Thai Red Cross Emerging Infectious Diseases Health Science Centre, King Chulalongkorn Memorial Hospital, Bangkok Thailand

## Abstract

**Background:**

Tau‐PET and plasma phosphorylated tau (p‐tau) have emerged as pivotal biomarkers for Alzheimer’s disease. Despite the practical advantages of using plasma p‐tau, there is limited understanding regarding its relationship with the topographic distribution of tau‐PET, particularly within the Southeast Asian population. This study aims to elucidate the correlation between plasma p‐tau levels and the spatial patterns observed in tau‐PET among Thai patients diagnosed with amnestic mild cognitive impairment (MCI) or mild dementia due to probable Alzheimer’s disease (AD).

**Methods:**

Thirty patients were included in this study. Correlations between plasma p‐tau and tau‐PET standardized uptake value ratio (SUVR) in each cortical region were analyzed. Patients were further stratified into low and high amyloid‐beta (Aβ) groups using Aβ centiloid (AβCL) median split for additional subgroup analyses. Plasma p‐tau217 was measured on the Mesoscale Discovery platform with the S‐PLEX Human Tau (pT217) Kit. PET tracers for tau and Aβ were PI‐2620 and 18F‐florbetaben. Brain parcellation for regional tau‐PET analyses was performed using FreeSurfer.

**Results:**

The mean age was 69.8 ± 8.4 years, with 20 female patients (66.7%). The median[IQR] value for plasma p‐tau217 was 7.87[11.37] pg/mL. Median[IQR] values for overall tau‐PET SUVR and AβCL were 1.20[0.35] and 43.56[53.70], respectively. Figure 1 illustrates cortical regions where plasma p‐tau217 significantly correlates with tau‐PET SUVR (p<0.05), with higher color intensity indicating stronger correlation coefficients. Cortical parcels demonstrating the highest correlation coefficients include the right anterior cingulate gyrus (r=0.74, p<0.001) and superior frontal gyri in both the left (r=0.73, p<0.001) and right (r=0.72, p<0.001) hemispheres. Figures 2 and 3 depict such cortical regions in low Aβ and high Aβ groups, respectively.

**Conclusions:**

In our study population, plasma p‐tau and tau‐PET SUVR exhibit correlations in most cortical regions, with a characteristic sparing of primary cortices. These correlations cluster in bilateral inferomedial temporal regions in the low Aβ group. In the high Aβ group, correlations are primarily observed in neocortical areas, including frontal, anterior temporal, and temporoparietal regions. This suggests that the relationship between plasma p‐tau and tau pathology may vary based on Aβ levels, providing valuable information for understanding disease progression.